# Analysis of the association between the acetabular morphology and femoral head in children aged 0–3 years with developmental hip dysplasia

**DOI:** 10.3389/fped.2023.1310411

**Published:** 2023-11-27

**Authors:** Liukun Xu, Bo Wang, Li Wang, Zhiqun Zhang

**Affiliations:** ^1^ Department of Orthopaedic Surgery, Children’s Hospital of Nanjing Medical University, Nanjing, China; ^2^ Department of Anesthesiology and Surgery, Children's Hospital of Nanjing Medical University, Nanjing, China

**Keywords:** developmental dysplasia of the hip, MRI, acetabulum, femoral head, correlation analysis

## Abstract

**Background:**

Magnetic resonance imaging (MRI) has been advocated as a routine examination for preoperative and postoperative assessment of Developmental Dysplasia of the Hip (DDH). However, there is limited research regarding the correlation between acetabulum and femoral head morphology using preoperative MRI measurements.

**Objective:**

To explore the correlation between acetabulum and femoral head morphology in children with DDH aged 0–3 years, using MRI measurements as indicators.

**Methods:**

A Retrospective Analysis of MRI Data from 172 Children Diagnosed with Developmental Dysplasia of the Hip (DDH) at Nanjing Medical University Affiliated Children's Hospital, spanning from January 2017 to January 2022. Measurements were taken to assess various parameters reflecting hip socket morphology as well as the development status of the femoral head and ossifying nucleus. The correlation between these factors was explored using Pearson correlation analysis and multiple-factor linear regression. Statistical analysis was conducted using SPSS 18.0 software.

**Results:**

Pearson correlation analysis revealed statistically significant associations between the length of the ossifying nucleus ratio and age(mo.), BAI, BCAD, CTAD, and CTAD. The height of the ossifying nucleus ratio displayed statistically significant correlations with age(mo.) and BTAD. The length of the femoral head ratio exhibited statistically significant correlations with CAI, BCEA, and BCAD. Furthermore, the height of the femoral head ratio demonstrated a statistically significant correlation with BCEA. After adjusting for age(mo.), BMI, BCEA, and CCEA, BPoAcet and CPoAcet was found to be correlated with the length of the ossifying nucleus ratio. Preoperatively, the CAI, BAxAcet, BPoAcet, CPoAcet, and BTAD were correlated with the height of ossifying nucleus ratio after correcting for age, BMI, BCEA, and CCEA.

**Conclusion:**

The measurement parameters of hip socket morphology on MRI are associated with femoral head development, making them potential predictive indicators for femoral head development in DDH patients. These findings offer valuable insights for clinical decisions regarding the timing and approach of surgery in patients with developmental hip dislocation.

## Introduction

Developmental Dysplasia of the Hip (DDH) refers to a series of anatomical anomalies in the relationship between the femoral head and the acetabulum during the developmental process, including acetabular dysplasia, subluxation of the hip joint, and dislocation of the hip joint (1). The reported incidence of DDH varies between 0.1% and 5%, depending on the study population, inclusion criteria, and diagnostic methods (2).The surgical treatment of pediatric DDH aims to restore the concentric relationship between the acetabulum and the femoral head, thereby promoting normal acetabular development (6). However, cases where both the acetabulum and the femoral head exhibit developmental dysplasia present greater challenges (35). Previous studies have indicated a strong interdependence between the development of the hip joint and the interaction between the acetabulum and the femoral head (3). Existing research has predominantly focused on primary femoral head abnormalities leading to acetabular dysplasia, with a limited number of studies exploring the influence of acetabular morphology on femoral head development (4, 5).MRI offers the advantage of clear visualization of soft tissues, cartilage, boney structures, and plays a crucial role in the assessment of DDH before and after treatment (22, 23, 24). This study aims to evaluate the correlation between acetabular and femoral head morphology in children aged 0–3 years with DDH using MRI.”

## Methods

### Study population

A total of 172 pediatric patients diagnosed with Developmental Dysplasia of the Hip (DDH) were collected from Nanjing Medical University Affiliated Children's Hospital between January 2017 and January 2022. Among these patients, there were 15 males and 157 females, with an average age of 18.20 ± 6.86 months.

Inclusion Criteria: Initial diagnosis made before the age of 36 months. Unilateral developmental dysplasia of the hip. Complete pre-treatment radiological data. Exclusion Criteria: Secondary hip dislocation due to conditions such as cerebral palsy, purulent hip joint arthritis, trauma, or multi-joint contractures. Lack of pre-treatment radiological data. Bilateral developmental dysplasia of the hip.

This study has obtained approval from the Ethics Committee of Nanjing Medical University Affiliated Children's Hospital, and informed consent was obtained from the legal guardians of all the patients.

### Imaging examination

A superconducting 1.5 T magnetic resonance imaging (MRI) scanner manufactured by GE Healthcare (model: GE SignaHde 1.5 T) was used for the imaging scans. The scanning parameters were as follows: a body coil was used, with the following sequence parameters: coronal and transverse T1-weighted imaging (T1WI) with a repetition time (TR) of 476–623 ms, echo time (TE) of 22–23 ms, a matrix size of 512 × 336, and a field of view (FOV) of 300 mm × 300 mm; fast spin echo (TSE) sequence with coronal T2-weighted imaging (T2WI) with a TR of 3,450–4,000 ms, TE of 88–95 ms, a matrix size of 512 × 336, and a FOV of 250 mm × 250 mm. The scanning range extended from the superior margin of the iliac wing to the middle-upper portion of the femur, with 1–2 excitations, a slice thickness of 2 mm, and an interslice gap of 0.1 mm.

### Measurement parameters

**Figure 1:**
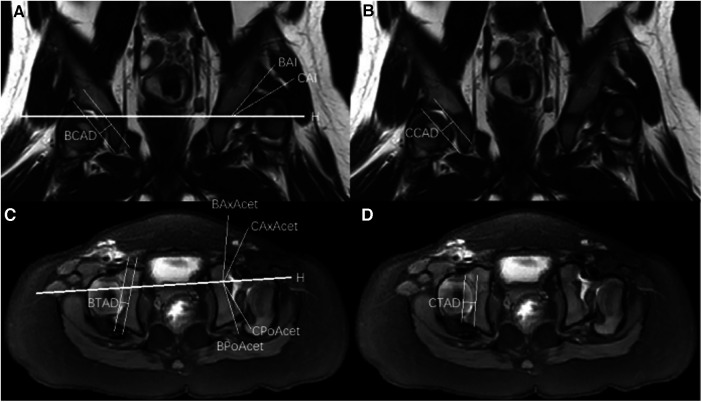
(**A**) Coronal T2-weighted magnetic resonance imaging demonstrating Hilgenreiner's line (H-H), boney acetabular index (BAI), cartilaginous acetabular index (CAI) and boney coronal acetabular depth (BCAD). (**B**) Coronal T2-weighted magnetic resonance imaging demonstrating cartilaginous coronal acetabular depth (CCAD). (**C**) Transverse T1-weighted magnetic resonance imaging demonstrating Boney axial acetabular angle (BAxAcet), cartilaginous axial acetabular angle (CAxAcet), boney posterior acetabular angle (BPoAcet), cartilaginous posterior acetabular angle (CPoAcet) and boney transverse acetabular depth (BTAD). (**D**) Transverse T1-weighted magnetic resonance imaging demonstrating cartilaginous transverse acetabular depth (CTAD).

The MRI images were analyzed and measured independently by two chief radiologists specialized in pediatric orthopedic radiology. The measurements were taken on the coronal and transverse T1-weighted imaging (T1WI) sequences at the level of the greatest extent of the femoral head epiphysis. The following measurements were performed to evaluate the acetabular morphology: Boney Acetabular Index (BAI), Cartilaginous Acetabular Index (CAI), Boney Center Edge Angle (BCEA), Cartilaginous Center Edge Angle (CCEA), Boney Coronal Acetabular Depth (BCAD), Cartilaginous Coronal Acetabular Depth (CCAD), Boney Axial Acetabular Angle (BAxAcet), Cartilaginous Axial Acetabular Angle (CAxAcet), Boney Posterior Acetabular Angle (BPoAcet), Cartilaginous Posterior Acetabular Angle (CPoAcet), Boney Transverse Acetabular Depth (BTAD), and Cartilaginous Transverse Acetabular Depth (CTAD) (Figure 1).

The measurements also included the length of the ossifying nucleus, the height of the ossifying nucleus, the length of the femoral head, and the height of the femoral head, all on the affected side and the unaffected side. The ratios between the affected and unaffected sides were used to assess the development of the femoral head.

### Statistical analysis

Statistical analysis was conducted using SPSS 18.0 software. Continuous variables were presented as mean ± standard deviation. Pearson linear correlation analysis was employed for the correlation analysis. Multiple linear regression analysis was used to examine the association between hip socket morphology imaging parameters and femoral head development while controlling for confounding factors. A *p*-value less than 0.05 was considered statistically significant.

## Results

### Characteristics of the study population

This study included 172 subjects, comprising 15 males and 157 females, with an average age of 18.20 ± 6.86 months and an average BMI of 17.39 ± 2.13. The shortest follow-up time was 1 year. All patients had a BAI of 37.83 ± 5.29°, CAI of 23.62 ± 5.21°, BCEA of −48.40 ± 22.41°, and CCEA of −39.26 ± 19.21°.

### Pearson correlation analysis

**Figure 2:**
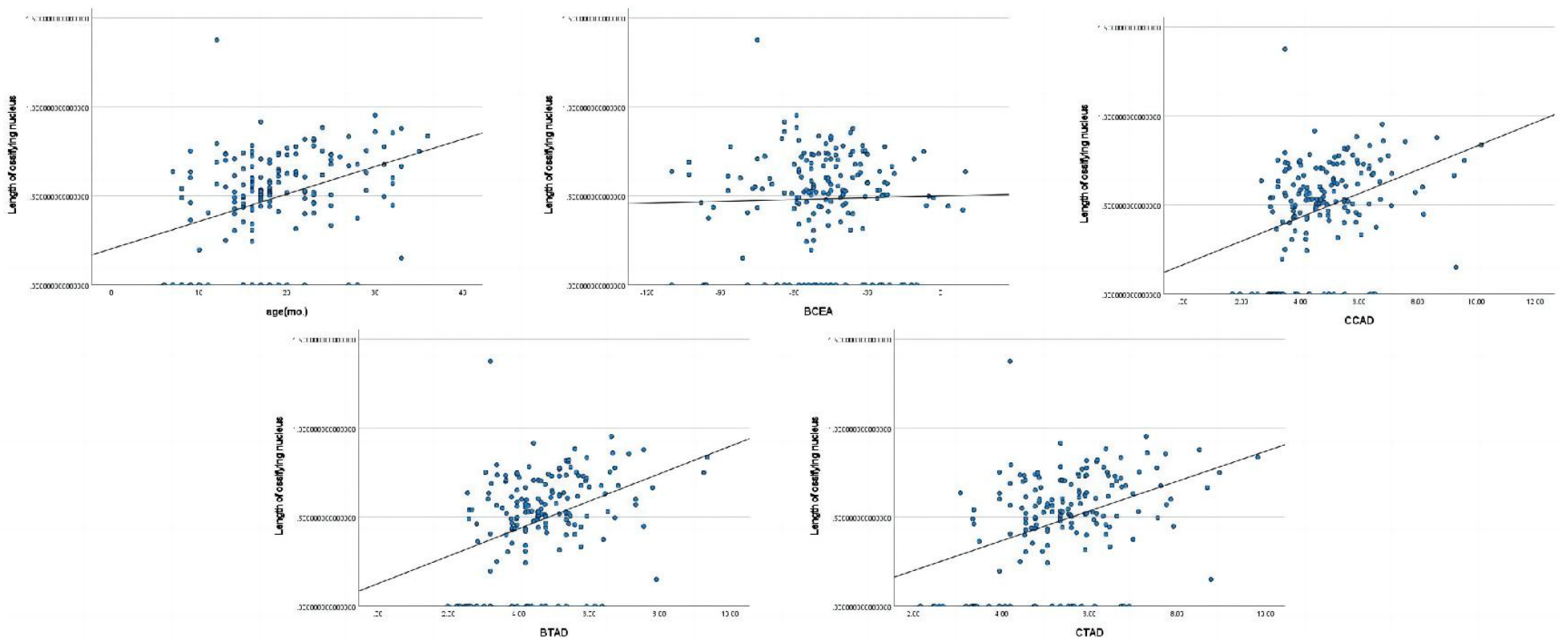
Pearson correlation analysis indicated statistically significant associations between the length of ossifying nucleus ratio and age(mo.), BAI, BCAD, CTAD, and CTAD.

**Figure 3:**
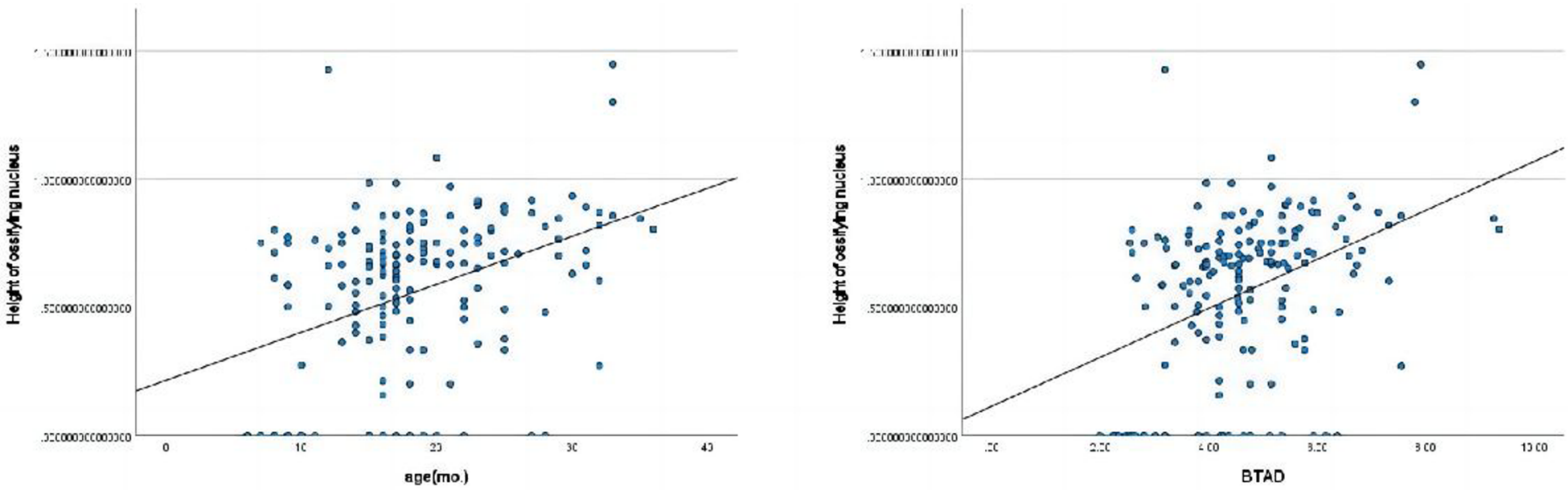
Pearson correlation analysis indicated statistically significant associations between the height of ossifying nucleus ratio and age(mo.), BTAD.

**Figure 4:**
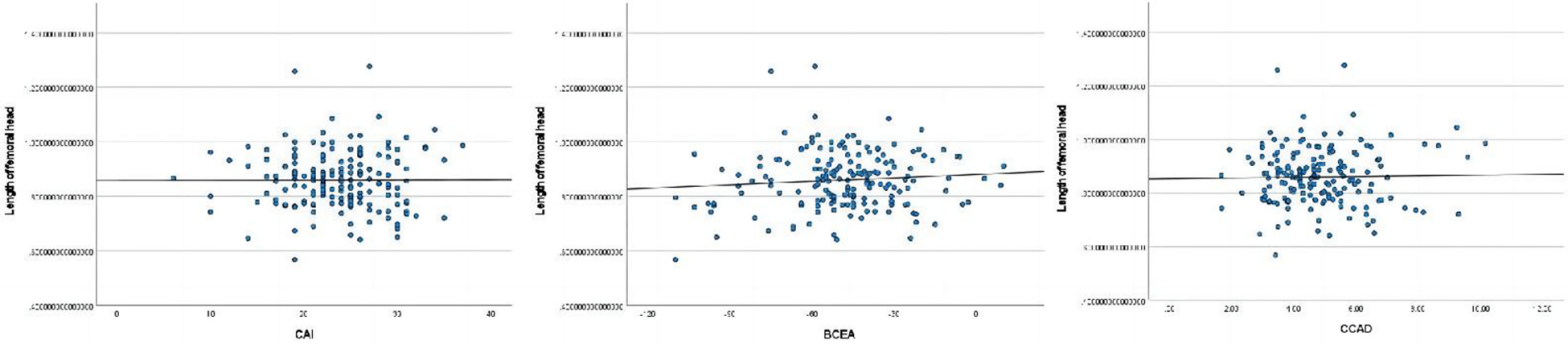
Pearson correlation analysis indicated statistically significant associations between the length of femoral head ratio and CAI, BCEA, BCAD.

**Figure 5:**
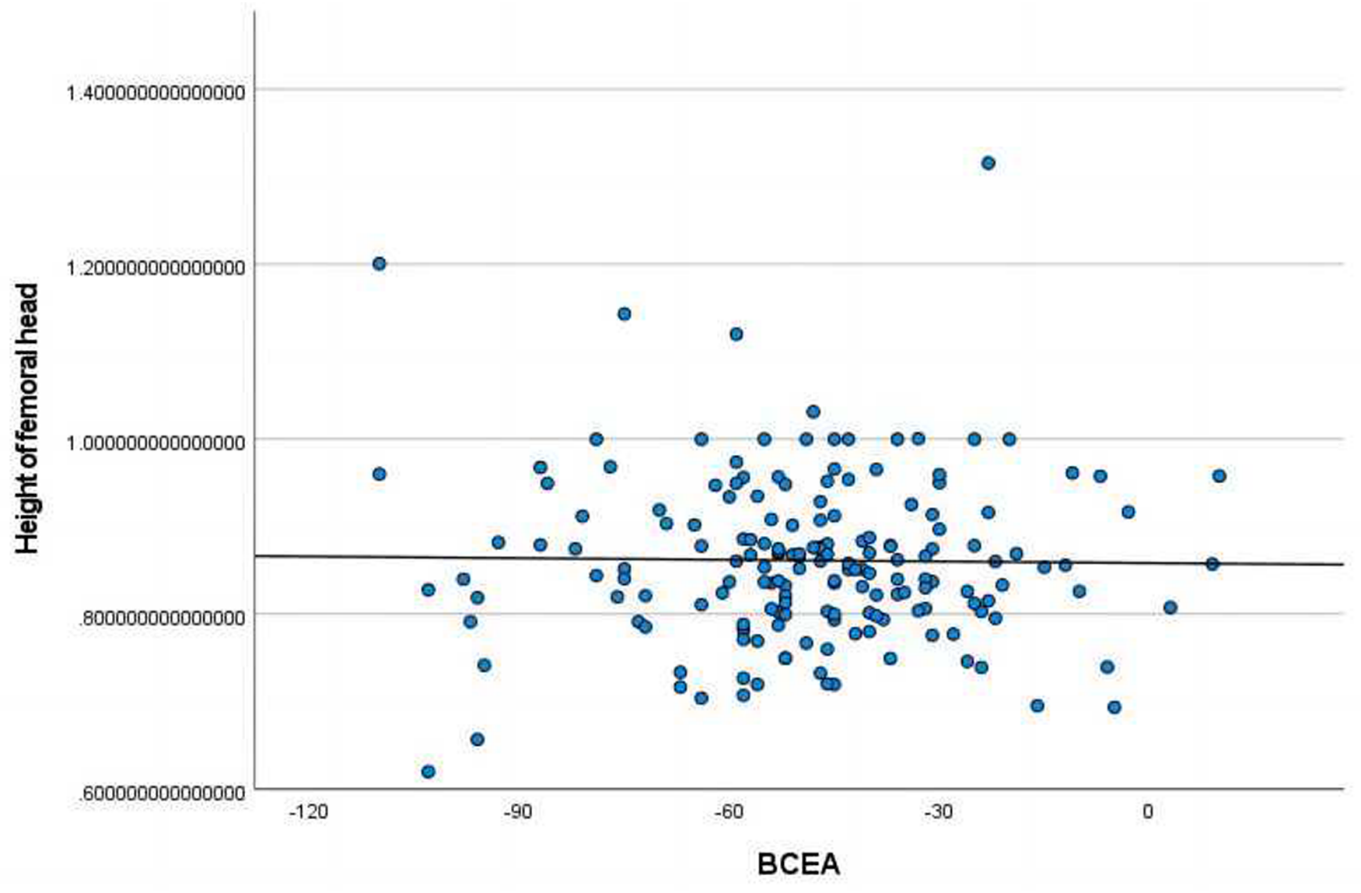
Pearson correlation analysis indicated statistically significant associations between the height of femoral head ratio and BCEA.

Pearson correlation analysis revealed statistically significant associations:

The length of ossifying nucleus ratio had significant correlations with age(mo.), BAI, BCAD, CTAD, and CTAD (Figure 2).
The height of ossifying nucleus ratio exhibited statistically significant correlations with age(mo.) and BTAD (Figure 3).
The length of femoral head ratio showed statistically significant correlations with CAI, BCEA, and BCAD (Figure 4).
The height of femoral head ratio displayed a statistically significant correlation with BCEA (Figure 5).

### Multiple linear regression

Multiple linear regression analysis demonstrated the following associations in models without adjusting for confounding factors: All of the measurements of the acetabular morphology were correlated with the length of ossifying nucleus ratio. After adjusting for age (mo.) and BMI, BAxAcet and CPoAcet were associated with the length of ossifying nucleus ratio. After adjusting for age (mo.), BMI, BCEA, and CCEA, BPoAcet and CPoAcet were related to the length of ossifying nucleus ratio (Table 1).

**Table 1:** Multiple linear regression analysis between the acetabular morphology and the length of ossifying nucleus ratio.

	Model 1^a^		Model 2^b^		Model^c^	
	β	*p* value	β	*p* value	β	*p* value
AI						
Q1	Ref.		Ref.		Ref.	
Q2	0.37	0.007	−0.93	0.357	−0.522	0.345
Q3	0.541	*<0*.*001*	0.466	0.289	0.43	0.342
Q4	0.512	*<0*.*001*	−0.495	0.368	−0.616	0.279
*p* for trend	<0.001		0.13		0.127	
CAI						
Q1	Ref.		Ref.		Ref.	
Q2	0.447	0.001	0.132	0.8	−0.013	0.981
Q3	0.453	0.002	−0.215	0.498	−0.281	0.392
Q4	0.425	0.008	−0.52	0.161	−0.69	0.081
*p* for trend	<0.001		0.311		0.075	
BCAD						
Q1	Ref.		Ref.		Ref.	
Q2	0.491	0.001	0.099	0.809	−0.474	0.468
Q3	0.478	*<0*.*001*	−0.408	0.371	−0.437	0.349
Q4	0.376	0.015	−0.161	0.566	−0.428	0.144
*p* for trend	<0.001		0.171		0.16	
CCAD						
Q1	Ref.		Ref.		Ref.	
Q2	0.486	0.001	0.059	0.836	−0.84	0.213
Q3	0.49	*<0*.*001*	1.419	0.055	−1.547	0.041
Q4	0.409	0.006	−0.138	0.618	−0.432	0.166
*p* for trend	<0.001		0.189		0.145	
BAxAcet						
Q1	Ref.		Ref.		Ref.	
Q2	0.525	*<0*.*001*	−0.001	0.999	−0.001	0.985
Q3	0.518	*<0*.*001*	−0.457	0.437	−0.515	0.402
Q4	0.437	0.001	−0.192	0.543	−0.381	0.455
*p* for trend	<0.001		0.271		0.212	
CAxAcet						
Q1	Ref.		Ref.		Ref.	
Q2	0.533	*<0*.*001*	0.114	0.885	0.196	0.82
Q3	0.492	*<0*.*001*	−0.712	0.222	−0.706	0.214
Q4	0.538	*<0*.*001*	−0.097	0.865	−0.12	0.837
*p* for trend	<0.001		0.159		0.144	
BPoAcet						
Q1	Ref.		Ref.		Ref.	
Q2	0.384	0.006	−0.083	0.872	−0.137	0.796
Q3	0.477	0.001	−0.283	0.474	−0.336	0.408
Q4	0.466	0.005	−0.067	0.887	−0.159	0.746
*p* for trend	<0.001		**0**.**02**		**0.015**	
CPoAcet						
Q1	Ref.		Ref.		Ref.	
Q2	0.604	*<0*.*001*	1.099	0.016	1.126	0.019
Q3	0.433	0.002	0.781	0.186	0.878	0.145
Q4	0.433	0.012	−0.326	0.6	−0.4	0.533
*p* for trend	<0.001		**0**.**025**		**0**.**019**	
BTAD						
Q1	Ref.		Ref.		Ref.	
Q2	0.384	0.006	−0.171	0.772	−0.208	0.729
Q3	0.553	*<0*.*001*	0.282	0.596	0.187	0.732
Q4	0.422	0.005	−0.343	0.263	−0.4	0.212
*p* for trend	<0.001		0.149		0.085	
CTAD						
Q1	Ref.		Ref.		Ref.	
Q2	0.525	*<0*.*001*	0.135	0.793	0.067	0.9
Q3	0.533	*<0*.*001*	0.1	0.867	0.008	0.896
Q4	0.442	0.003	−0.329	0.325	−0.38	0.274
*p* for trend	<0.001		0.25		0.188	

^a^ Without adjusting for confounding factors.

^b^ Adjusting for age (mo.) and BMI.

^c^ Adjusting for age (mo.), BMI, BCEA, and CCEA.

The bold value means that there is an association between these indicators of acetabular morphology and femoral head development.

Without adjusting for confounding factors, all of the measurements of the acetabular morphology were correlated with the height of ossifying nucleus ratio. After adjusting for age (mo.) and BMI, CAI, BAxAcet, BPoAcet, and CPoAcet were correlated with the height of ossifying nucleus ratio. After adjusting for age (mo.), BMI, BCEA, and CCEA, CAI, BAxAcet, BPoAcet, CPoAcet, and BTAD were related to the height of ossifying nucleus ratio (Table 2).

**Table 2:** Multiple linear regression analysis between the acetabular morphology and the height of ossifying nucleus ratio.

	Model 1^a^		Model 2^b^		Model 3^c^	
	β	*p* value	β	*p* value	β	*p* value
AI						
Q1	Ref.		Ref.		Ref.	
Q2	0.387	0.005	−0.441	0.405	−0.466	0.4
Q3	0.457	0.006	0.158	0.729	0.14	0.459
Q4	0.466	0.002	−0.824	0.139	−0.909	0.117
*p* for trend	<0.001		0.05		0.054	
CAI						
Q1	Ref.		Ref.		Ref.	
Q2	0.445	0.002	−0.158	0.759	−0.623	0.626
Q3	0.44	0.003	−0.245	0.442	−0.29	0.381
Q4	0.402	0.012	−0.476	0.212	−0.612	0.137
*p* for trend	<0.001		**0**.**023**		**0**.**018**	
BCAD						
Q1	Ref.		Ref.		Ref.	
Q2	0.453	0.004	−0.756	0.247	−0.77	0.251
Q3	0.448	0.002	−0.571	0.215	−0.585	0.215
Q4	0.379	0.014	−0.299	0.297	−0.345	0.251
*p* for trend	<0.001		0.051		0.052	
CCAD						
Q1	Ref.		Ref.		Ref.	
Q2	0.46	0.002	−1.012	0.126	−1.057	0.122
Q3	0.475	0.001	−1.665	0.024	−1.76	0.02
Q4	0.43	0.004	−0.233	0.441	−0.286	0.388
*p* for trend	<0.001		0.122		0.11	
BAxAcet						
Q1	Ref.		Ref.		Ref.	
Q2	0.481	0.55	0.355	−0.542	0.376	
Q3	0.462	0.003	−1.031	0.081	−1.007	0.083
Q4	0.452	0.004	−0.395	0.424	−0.508	0.333
*p* for trend	<0.001		**0**.**04**		**0**.**034**	
CAxAcet						
Q1	Ref.		Ref.		Ref.	
Q2	0.518	*<0.001*	0.116	0.885	0.073	0.933
Q3	0.469	0.002	−1.006	0.083	−0.997	0.099
Q4	0.501	0.001	−0.309	0.596	−0.329	0.583
*p* for trend	<0.001		0.05		0.051	
BPoAcet						
Q1	Ref.		Ref.		Ref.	
Q2	0.363	0.01	−0.567	0.267	−0.594	0.258
Q3	0.446	0.003	−0.491	0.214	−0.52	0.203
Q4	0.385	0.024	−0.39	0.425	−0.461	0.366
*p* for trend	<0.001		**0**.**002**		**0**.**002**	
CPoAcet						
Q1	Ref.		Ref.		Ref.	
Q2	0.564	*<0.001*	0.692	0.14	0.741	0.136
Q3	0.43	0.002	0.487	0.409	0.532	0.381
Q4	0.375	0.032	−0.683	0.277	−0.723	0.271
*p* for trend	<0.001		**0**.**004**		**0**.**003**	
BTAD						
Q1	Ref.		Ref.		Ref.	
Q2	0.383	0.007	−0.433	0.459	−0.462	0.44
Q3	0.475	0.003	−0.19	0.731	−0.256	0.656
Q4	0.433	0.004	−0.263	0.403	−0.297	0.361
*p* for trend	<0.001		0.052		***0***.***036***	
CTAD						
Q1	Ref.		Ref.		Ref.	
Q2	0.475	0.002	−0.371	0.475	−0.491	0.435
Q3	0.511	*<0.001*	−0.77	0.777	−0.169	0.784
Q4	0.454	0.002	−0.21	0.534	−0.257	0.467
*p* for trend	<0.001		0.136		0.12	

^a^ Without adjusting for confounding factors.

^b^ Adjusting for age (mo.) and BMI.

^c^ Adjusting for age (mo.), BMI, BCEA, and CCEA.

The bold value means that there is an association between these indicators of acetabular morphology and femoral head development.

Without adjusting for confounding factors, all of the measurements of the acetabular morphology were not correlated with the length of femoral head ratio.After adjusting for age (mo.) and BMI, all of the measurements of the acetabular morphology were not correlated with the length of femoral head ratio.After adjusting for age (mo.), BMI, BCEA, and CCEA, CAxAcet were related to the length of femoral head ratio (Table 3).

**Table 3:** Multiple linear regression analysis between the acetabular morphology and the length of femoral head ratio.

	Model 1^a^		Model 2^b^		Model 3^c^	
	β	*p* value	β	*p* value	β	*p* value
AI						
Q1	Ref.		Ref.		Ref.	
Q2	−0.086	0.543	0.025	0.966	0.093	0.875
Q3	0.069	0.693	0.291	0.572	0.179	0.723
Q4	0.056	0.731	−0.2	0.764	−0.49	0.449
*p* for trend	0.592		0.115		0.062	
CAI						
Q1	Ref.		Ref.		Ref.	
Q2	0.001	0.996	0.213	0.53	−0.106	0.853
Q3	0.058	0.712	0.341	0.364	0.194	0.602
Q4	0.058	0.731	−0.17	0.702	−0.89	0.277
*p* for trend	0.802		0.432		0.235	
BCAD						
Q1	Ref.		Ref.		Ref.	
Q2	0.069	0.675	−0.334	0.667	−0.372	0.618
Q3	0.062	0.686	0.192	0.723	0.101	0.846
Q4	−0.01	0.95	−0.165	0.624	−0.311	0.346
*p* for trend	0.602		0.141		0.071	
CCAD						
Q1	Ref.		Ref.		Ref.	
Q2	0.01	0.951	−0.89	0.253	−1.085	0.148
Q3	0.019	0.904	−0.809	0.367	−10,100	0.206
Q4	−0.081	0.605	0.046	0.889	−0.53	0.132
*p* for trend	0.871		0.643		0.413	
BAxAcet						
Q1	Ref.		Ref.		Ref.	
Q2	0.061	0.695	0.366	0.599	0.35	0.603
Q3	0.089	0.583	0.231	0.745	0.182	0.794
Q4	0.093	0.577	0.109	0.85	−0.218	0.703
*p* for trend	0.842		0.526		0.284	
CAxAcet						
Q1	Ref.		Ref.		Ref.	
Q2	0.01	0.947	−1.23	0.106	−1.252	0.186
Q3	−0.003	0.984	−0.18	0.797	−0.061	0.929
Q4	0.049	0.763	−0.32	0.642	−0.39	0.554
*p* for trend	0.552		0.086		**0**.**04**	
BPoAcet						
Q1	Ref.		Ref.		Ref.	
Q2	−0.038	0.797	0.172	0.76	0.099	0.865
Q3	−0.017	0.911	−0.227	0.63	−0.343	0.456
Q4	0.094	0.598	−0.302	0.58	−0.5	0.346
*p* for trend	0.674		0.19		0.095	
CPoAcet						
Q1	Ref.		Ref.		Ref.	
Q2	0.065	0.677	0.619	0.267	0.547	0.329
Q3	−0.03	0.839	0.321	0.62	0.48	0.444
Q4	0.183	0.308	0.652	0.347	0.511	0.45
*p* for trend	0.651	0.17	0.096			
BTAD						
Q1	Ref.		Ref.		Ref.	
Q2	−0.075	0.61	−0.437	0.496	−0.548	0.374
Q3	0.083	0.626	0.425	0.508	0.214	0.735
Q4	−0.065	0.681	−0.322	0.386	0.419	0.214
*p* for trend	0.974		0.893		0.594	
CTAD						
Q1	Ref.		Ref.		Ref.	
Q2	0.034	0.833	−0.132	0.828	−0.344	0.56
Q3	0.011	0.945	0.005	0.995	−0.031	0.964
Q4	−0.086	0.585	−0.494	0.231	−0.638	0.101
*p* for trend	0.834		0.53		0.297	

^a^ Without adjusting for confounding factors.

^b^ Adjusting for age (mo.) and BMI.

^c^ Adjusting for age (mo.), BMI, BCEA, and CCEA.

The bold value means that there is an association between these indicators of acetabular morphology and femoral head development.

Within the three models, all of the measurements of the acetabular morphology were not correlated with the height of femoral head ratio (Table 4).

**Table 4:** Multiple linear regression analysis between the acetabular morphology and the height of femoral head ratio.

	Model 1^a^		Model 2^b^		Model 3^c^	
	β	*p* value	β	*p* value	β	*p* value
AI						
Q1	Ref.		Ref.		Ref.	
Q2	−0.092	0.519	−0.361	0.535	−0.442	0.467
Q3	0.059	0.736	−0.489	0.355	−0.486	0.361
Q4	0.004	0.979	−0.224	0.712	−0.226	0.745
*p* for trend	0.697		0.119		0.12	
CAI						
Q1	Ref.		Ref.		Ref.	
Q2	−0.032	0.830	−0.687	0.231	−0.748	0.217
Q3	0.015	0.922	0.314	0.342	0.094	0.811
Q4	0.005	0.974	−0.484	0.271	−0.539	0.26
*p* for trend	0.726		0.145		0.129	
BCAD						
Q1	Ref.		Ref.		Ref.	
Q2	0.106	0.521	−0.12	0.877	−0.124	0.876
Q3	0.102	0.506	0.244	0.448	0.632	0.248
Q4	−0.151	0.347	−0.511	119	−0.529	0.126
*p* for trend	0.596		0.061		0.059	
CCAD						
Q1	Ref.		Ref.		Ref.	
Q2	−0.025	0.877	−0.774	0.315	−0.762	0.34
Q3	0.008	0.959	0.323	0.315	−0.499	0.589
Q4	−0.058	0.712	−0.281	0.427	−0.29	0.438
*p* for trend	0.955		0.577		0.555	
BAxAcet						
Q1	Ref.		Ref.		Ref.	
Q2	0.031	0.841	−0.122	0.86	0.115	0.873
Q3	0.074	0.648	0.578	0.41	0.646	0.384
Q4	0.031	0.852	−0.473	0.405	−0.499	0.411
*p* for trend	0.746		0.177		0.16	
CAxAcet						
Q1	Ref.		Ref.		Ref.	
Q2	−0.392	0.7	−0.3	0.745	−0.392	0.7
Q3	0.034	0.826	0.207	0.765	0.214	0.767
Q4	−0.008	0.959	−0.445	0.514	−0.447	0.525
*p* for trend	0.688		0.116		0.113	
BPoAcet						
Q1	Ref.		Ref.		Ref.	
Q2	−0.012	0.934	−0.091	0.871	−0.093	0.872
Q3	0.025	0.872	−0.027	0.954	−0.005	0.991
Q4	0.075	0.673	−0.748	0.162	−0.782	0.164
*p* for trend	0.632		0.059		0.055	
CPoAcet						
Q1	Ref.		Ref.		Ref.	
Q2	0.023	0.882	−0.211	0.705	−0.191	0.747
Q3	0.023	0.873	0.447	0.489	0.458	0.492
Q4	0.16	0.374	−0.127	0.854	−0.107	0.883
*p* for trend	0.703		0.116		0.111	
BTAD						
Q1	Ref.		Ref.		Ref.	
Q2	−0.031	0.835	−0.434	0.498	−0.439	0.504
Q3	0.095	0.574	−0.299	0.639	−0.297	0.656
Q4	0.011	0.946	0.055	0.882	0.061	0.974
*p* for trend	0.961		0.763		0.7	
CTAD						
Q1	Ref.		Ref.		Ref.	
Q2	−0.001	0.996	0.039	0.948	0.061	0.922
Q3	0.033	0.832	0.017	0.981	0.032	0.965
Q4	−0.06	0.7	−0.362	0.359	−0.373	0.368
*p* for trend	0.893		0.422		0.395	

^a^ Without adjusting for confounding factors.

^b^ Adjusting for age (mo.) and BMI.

^c^ Adjusting for age (mo.), BMI, BCEA, and CCEA.

## Discussion

The hip joint is among the most intricate articulations of the human body, comprising the acetabulum, the proximal femur, and the connecting soft tissues. In children, the hip socket is divided into three parts, formed by the connection of the ischium, pubis, and ilium through the Y-shaped cartilage. The development of the acetabulum is closely intertwined with that of the femoral head, as when they fail to make contact, the acetabulum assumes a flattened shape (6). At birth, the femoral head is entirely composed of cartilaginous tissue, and the appearance of the ossification center of the femoral head occurs around the age of six months. As age progresses, the anteversion angle and neck-shaft angle of the femur decrease. The growth and development of the hip joint are contingent upon the concentric alignment of the femoral head within the acetabulum and the harmonious growth of the Y-shaped cartilage and the acetabular cartilage. Any alteration in either of these factors can potentially lead to the occurrence of developmental dysplasia of the hip (DDH) (7,8).

Previous studies have indicated that the development of the hip joint heavily relies on the interaction between the acetabulum and the femoral head. The acetabulum requires a spherical femoral head as a growth template, while the spherical growth of the femoral head and the symmetrical development of the epiphysis also necessitate the coverage of the acetabulum (3, 9). Existing research findings have primarily focused on the primary deformity of the femoral head leading to acetabular dysplasia (10–16). When hip dislocation occurs and the femoral head fails to make contact with the acetabulum, the acetabulum assumes a flattened shape. An animal study demonstrated that removal of the femoral head in rats results in inadequate development of the acetabulum (12). Complete absence of the proximal femur in humans leads to acetabular deficiency (17). In cases of unstable concentric reduction, hip joint dislocation in children of walking age can result in acetabular “saucerization” (18). Only a few studies have investigated the impact of acetabular morphology on the development of the femoral head. S.D. Steppacher et al. found that in cases of dysplastic hip joints, there is a decreased acetabular depth, along with an elliptical shape of the femoral head, reduced epiphyseal height, and asymmetric extension of the epiphysis on both sides, indicating that different acetabular coverage affects the anatomical morphology of the femoral head (3). Wudbhav N. studied the sphericity of the femoral head in patients with developmental dysplasia of the hip (DDH) and found that the affected hips had lower femoral head sphericity scores compared to the unaffected hips, with no correlation to age (19). Due to anatomical abnormalities and physical factors, the development of the affected hip joint in DDH patients does not correspond to that of the unaffected side. Research suggests that the magnitude of stress load is inversely related to the rate of epiphyseal growth, a pattern applicable to both the acetabulum and the femoral head epiphysis (20).

The objective of this study is to observe the development of the acetabulum and femoral head in children aged 0–3 years with unilateral Developmental Dysplasia of the Hip (DDH) and to explore the relationship between them. To eliminate individual differences, we used the ratio of measurements on the affected side to those on the unaffected side to represent the development of the femoral head. We measured various indicators on MRI that reflect acetabular morphology, which to some extent can reflect the primary acetabular dysplasia in DDH patients.

MRI offers clear visualization of non-ossified femoral heads, acetabular cartilage, acetabular labrum, and fibrous adipose tissue distribution within the acetabular socket (22, 23). It has been widely used for preoperative planning and postoperative evaluation of surgical treatment for developmental hip dysplasia, and some studies have employed it for monitoring joint cartilage growth in children's growth and development (24). Pearson correlation analysis suggests a higher correlation between hip socket morphology and ossifying nucleus development. This aligns with previous research findings, which suggest that the development of the ossifying nucleus is more susceptible to stress-induced ischemic injury (21). Age, BMI, and CCEA may act as confounding factors. Whether in a physiological or pathological context, the ossifying nucleus has developmental potential, and CEA, to some extent, represents the degree of hip joint dislocation. In cases of subluxation, there is abnormal stress between the femoral head and the outer edge of the acetabulum. Fully dislocated femoral heads are exposed to an extremely abnormal developmental environment, and excessive body weight in walking-age children can increase the load on the hip joint. Therefore, we corrected for these three factors in our multiple linear regression analysis. Multiple linear regression analysis reveals an association between hip socket morphology and femoral head development. After adjusting for confounding factors, we found correlations between boney posterior acetabular angle (BPoAcet) and cartilaginous posterior acetabular angle (CPoAcet) with the ossifying nucleus transverse diameter ratio. This may be related to the direction of force in the hip joint, which experiences varying stress directions in different states, with the posterior aspect of the acetabulum bearing more load during walking (25). We observed that factors influencing ossifying nucleus height were more varied. After adjusting for age (mo.) and BMI, CAI, BAxAcet, BPoAcet, CPoAcet, and BTAD were related to the height of ossifying nucleus ratio. As children with dislocated femoral heads experience abnormal stresses, the stress directions from the outer and upper aspects of the acetabulum are most variable. According to Wolff's law (34), bone trabeculae adapt to changes in the mechanical environment, and ossifying nucleus height is more sensitive to changes in stress in the context of DDH. Reduced acetabular depth implies inadequate coverage of the femoral head, resulting in changes in the position and size of the femoral head load, which affects the development of the ossifying nucleus (5). After correcting for all confounding factors, only CAxAcet was related to the femoral head transverse diameter ratio. This might be an expression of continuous stress stimulation leading to thickening of acetabular cartilage. With advancing age, long-term changes in the developmental environment will gradually affect the femoral head's response to stress changes. Long-term changes in the developmental environment disrupt the balance of apoptosis, significantly impacting the normal development of cartilage and ossification (26). In DDH, the acetabulum is smaller than normal, and the peak stresses on the hip joint are significantly higher, leading to joint cartilage damage and directly affecting the development of the femoral head size. As age increases, the remodeling capacity of the acetabulum and femoral head decreases (27, 28). Continuous subluxation increases the load on the outer edge of the acetabulum, inhibiting the development and ossification of the acetabulum's outer edge. Stress concentrates on the complex formed by the labrum and acetabular rim on the outer-upper aspect of the hip joint, resulting in continuous cartilage damage to the femoral head and acetabulum (29–32). Continuous dislocation leads to the absence of a spherical structure of the femoral head as a growth template for the acetabulum, causing the acetabulum to develop into a flatter shape.

This study leans towards the idea that acetabular dysplasia is the initial pathology of DDH, and changes in the femoral head are secondary adaptive phenomena (33). Although there is no direct evidence, we believe that even in the absence of femoral head necrosis, abnormal femoral head morphology can have a detrimental effect on hip joint development, and the morphological abnormalities of the femoral head are often difficult to correct through surgery. Additionally, femoral head development cannot be effectively monitored in the early stages using simple methods such as x-ray. Therefore, identifying reliable acetabular morphology indicators for assessing femoral head development is crucial.

The surgical focus of pediatric DDH remains on acetabular osteotomy and various procedures to improve acetabular coverage, restoring the concentric relationship between the acetabulum and the femoral head and stabilizing it. Some studies (35) have focused on long-term changes in the proximal femoral morphology in DDH, with findings suggesting that the femoral neck length decreases as the severity of the disease increases and tends to incline forward. Notably, there are significant differences in femoral head anterior tilt between male and female DDH patients. Apart from the common complication of femoral head avascular necrosis, femoral head deformity also requires attention during the treatment of DDH.

This study has several limitations. Although we evaluated acetabular morphology from different perspectives on MRI images, the measurements are still two-dimensional, while the complex relationship between the acetabulum and the femoral head should ideally be visualized in three dimensions. Femoral head morphology needs to be comprehensively assessed from a three-dimensional perspective. This study evaluated femoral head morphology in two dimensions, measuring its transverse diameter and width. Additionally, the sample size in this study is relatively small, and it is necessary to conduct multicenter studies to increase the sample size. This cross-sectional study only provides a preliminary exploration of the relationship between acetabular morphology and femoral head development, serving as a foundation for more in-depth mechanistic research. Continuous follow-up is required to observe long-term changes in femoral head morphology.

In conclusion, through a retrospective analysis of imaging data from 172 children with unilateral developmental hip dislocation aged 0–3 years, we found correlations between boney posterior acetabular angle, cartilaginous posterior acetabular angle, preoperative cartilaginous acetabular index, boney axial acetabular angle, boney transverse acetabular depth, and cartilaginous axial acetabular index with femoral head development. These factors may serve as predictive indicators for femoral head development in DDH patients, providing insights for clinical decision-making regarding the timing and method of surgery for developmental hip dislocation patients.

## Data Availability

The raw data supporting the conclusions of this article will be made available by the authors, without undue reservation.
